# Interaction of Intestinal Microorganisms with the Human Host in the Framework of Autoimmune Diseases

**DOI:** 10.3389/fimmu.2015.00594

**Published:** 2015-11-20

**Authors:** Borja Sánchez, Arancha Hevia, Sonia González, Abelardo Margolles

**Affiliations:** ^1^Department of Microbiology and Biochemistry of Dairy Products, Instituto de Productos Lácteos de Asturias, Consejo Superior de Investigaciones Científicas, Villaviciosa, Spain; ^2^Department of Functional Biology, University of Oviedo, Oviedo, Spain

**Keywords:** intestinal microbiota, systemic lupus erythematosus, molecular mimicry, toll-like receptors, dietary intervention

## Abstract

Autoimmune diseases, such as systemic lupus erythematosus (SLE), are caused by a complex interaction of environmental-, genetic-, and sex-related factors. Although SLE has traditionally been considered independent from the microbiota, recent work published during the last 5 years suggests a strong connection between SLE and the composition of our gut commensals as one of the main environmental factors linked to this disease. Preliminary data have evidenced that (i) interaction of certain microbial-derived molecules with specific cell receptors and (ii) the influence of certain commensal microorganisms over specific immune cell subsets plays an important role in the pathogenesis of SLE and SLE-like diseases. In addition, epigenetic changes driven by certain microbial groups have been recently proposed as an additional link between gut microbiota and SLE. As immune responses elicited against commensal bacteria are deeply dependent on the composition of the latter, and as microbial populations can be modified by dietary interventions, identifying the precise gut microorganisms responsible for worsening the SLE symptoms is of crucial importance for this and other SLE-related diseases, including antiphospholipid syndrome or lupus nephritis. In this minireview, the current knowledge on the relationships between microbes and SLE and SLE-related diseases is compiled and discussed.

## Gut Microbiota and Immune System

The term human microbiota describes the ensemble of microorganisms inhabiting our body, which account for 10 times more cells than our own cells, with the colon being the place in the body populated by the largest number of microbes. Alterations of gut microbial populations (also termed intestinal dysbiosis) have been associated with a large number of autoimmune and chronic inflammatory diseases during the last 10 years, for instance, rheumatoid arthritis, type 1 diabetes, inflammatory bowel disease, and recently systemic lupus erythematosus (SLE) (1, 2).

Our microbiota and immune system have co-evolved in a symbiotic way; while bacteria helps in digestion, presence of certain commensals triggers different signals that drive proper maturation of our immune system. In turn, the colon provides the right environment to host these populations. Microbiota composition can be quickly modified by diet or by the use of certain living bacteria, known as probiotics. Intestinal microbiota influences the development of autoimmune diseases, and so this influence might be modulated through specific diets or with the administration of specific probiotic strains (3). Scientific evidence suggests that every type of microorganism might have a specific mechanism of action over the host immune system, balancing anti- and pro-inflammatory responses given some host factors, such as the genetic background, sex, and the relative abundance of a given microorganism in the whole microbiota (4).

In the last years, some studies have identified different microbial molecules, notably located at the surface level, able to drive those immunological effects, among which extracellular polysaccharides and proteins, peptides, and teichoic acids are noteworthy (5). The aim of the present minireview is to compile and discuss current evidence of the potential influence of gut microbes on SLE pathogeny and other related autoimmune diseases.

## Systemic Lupus Erythematosus as a Model Autoimmune Disease

Systemic lupus erythematosus is a complex disease with remarkable heterogeneity in its clinical features, which includes malar rash, photosensitivity, and renal disorders among others. About half of the patients develop renal symptoms within the first year, and in fact kidney-specific molecules are the main target for immunosuppressive treatments. From the immunological point of view, SLE is characterized by aberrant responses at different levels, including uncontrolled T-cell differentiation and activation, abnormal polyclonal B-cell activation/proliferation, and autoantibody (IgGs) production linked to immune complex formation (6).

The key immunological characteristic of SLE is B-cell hyperactivity, which results in the production of a wide set of autoantibodies recognizing more than 100 different ligands, such as nuclear DNA or histones, ribonucleoproteins, or cardiolipin (7). Binding of these autoantibodies to self-antigens induces the formation of immune complexes, whose accumulation triggers molecular inflammation and leads to severe damages due to immunological clearance. For instance, injuries at the glomerular level are responsible for lupus nephritis (LN), the severity of the symptoms correlating with the autoantibody titers and, therefore, with the amount of immune complexes formed (8).

The SLE diagnosis cannot be done only on a serological basis, in spite of the abundant array of autoantibodies generated during its onset and development. SLE is diagnosed when 4 out of 11 specific criteria are fulfilled, the presence of elevated autoantibody titers representing only two of them (9). As B-cell hyperactivity underlies this auto-antigen generation, this cell type is target for several treatments, including monoclonal antibodies binding to specific B-cell-surface antigens (CD19, CD20, CD22) to trigger apoptosis/lysis, or peptides/proteins blocking binding of B-cell activating factors to B-cell receptors (BLyS, APRIL), thus, aborting maturation and proliferation (10). Remarkably, genetic susceptibility of the B-cell receptor gene is a factor favoring induction of B-cell hyperactivity by external stimuli and, therefore, conditioning an individual to autoimmunity (11).

In general, total IgG-class antibody titers in plasma were shown to be doubled in a SLE cohort regarding the controls (12). As it has been shown that specific IgGs raised against the gut microbiota displayed lower titers during active SLE episodes compared to inactive periods, it is conceivable to hypothesize that these specific antibodies are sequestered in immune complexes, contributing to the pathology of the disease, and opening the door to the involvement of certain bacteria at least in SLE relapses (12).

## Autoimmune Diseases, a Role for Gut Microbiota

The term “autoimmune disease” denotes a disorder where the immune system recognizes self-molecules as foreign, a process in which microbes may have a direct or indirect influence. Direct mechanisms involve growing and temporal persistence of the bacterium within the gut, as well as the secretion or release of different molecular mediators. On the contrary, indirect mechanisms involve the production of epigenetic changes in human cells driven by the presence of the bacterium. For these reasons, the relationship and interactions among commensal microbes, immune system, and epigenetics are an emerging field of great interest in autoimmune disease research (13).

It should be highlighted that host sex conditions the populations inhabiting our gut, which may condition the severity of the symptoms, as in SLE (14). In turn, the configuration of host immunity at both innate and adaptive levels may condition relative abundances within those gut commensal populations, establishing a complicated feedback between relative microorganism abundances, host sex and the way to shape the type of immune responses against self and foreign antigens (15).

Commensal microbiota composition has a deep effect in terms of immunomodulation, whatever the immunological context of the host. This statement is very well illustrated by a single bacterial species, the segmented filamentous bacteria (SFB), and might be applicable to every bacterial population. SFB have the ability to expand the set of Th17 cells, which are involved in many human autoimmune diseases (16). The Th17 response is pathogenic in certain animal models of autoimmune diseases such as rheumatoid arthritis or certain IBD types, but protective in others such as type 1 diabetes (17–19), so the specific effect of a single bacterium can be either positive or negative, depending on the immunological status of the host. Moreover, SFB effect varies depending on the host sex, and so Th17 stimulation confers protection in females but not in males in certain murine models of autoimmune diseases (18, 19).

**Figure 1:**
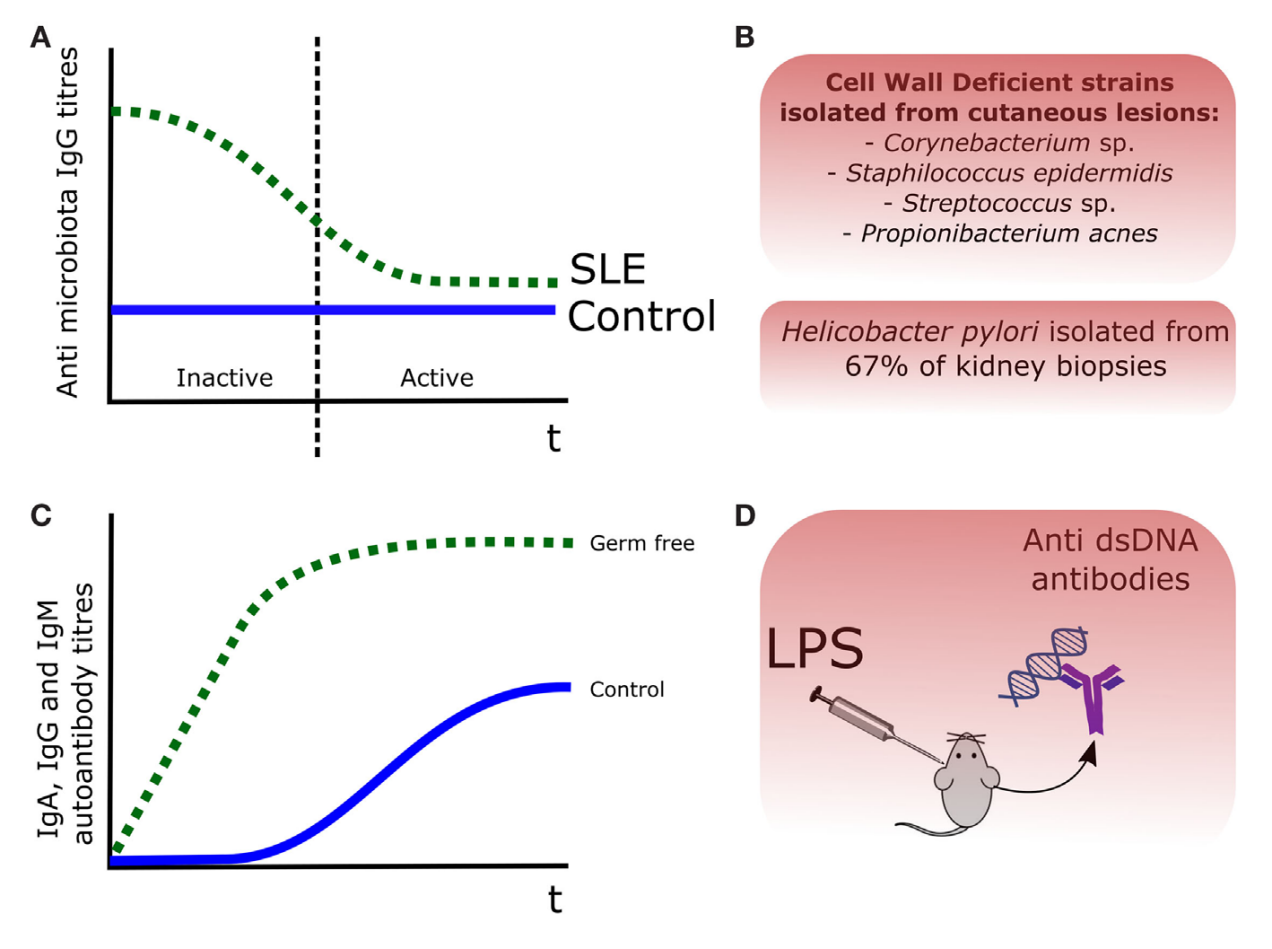
**Evidences of the role of the commensal microbiota in SLE pathogenesis**. **(A)** Antibody titers were increased in SLE patients, being those recognizing the gut microbiota decreased during the disease episodes, probably after binding to certain bacterial members (12). **(B)** Cell wall deficient forms (CWD) of commensal bacteria were isolated from skin lesions in LE patients (20), and *Helicobacter pylori* was isolated in the 67% of kidney biopsies from a LN cohort (25). **(C)** In the pristane animal model of SLE, production of autoantibodies was lower and delayed, in germ-free versus control mice (27). **(D)** Injection of LPS in mice induced production of anti-dsDNA antibodies (22).

Among the different autoimmune diseases, SLE has been considered independent from the microbiota, with few scientific evidences dating back to the 1970s and 1980s. If we examine carefully the scientific literature, we can find some evidences, suggesting a link between the presence of certain bacteria and SLE. For instance, cell wall deficient forms (CWD) of *Propionibacterium acnes*, *Corynebacterium* sp., *Staphylococcus epidermis*, and *Streptococcus* sp. were isolated in cutaneous lesions of patients affected by SLE and other SLE-related disorders such as cutaneous lupus erythematosus (LE) (20). Perhaps the strongest evidence linking a bacterial antigen and the production of self-antibodies in SLE-related murine models is lipopolysaccharide (LPS) (21). Injection of LPS in mice induced production of anti-ds DNA antibodies, which were associated with an increased formation of immune complexes in kidneys and an exacerbation of the LN symptoms, including glomerular dysfunction and chronic kidney dysfunction (22). Regarding LPS and the generation of autoantibodies in murine SLE models, two studies dating from the mid-1970s are available (23, 24). The situation has drastically changed during the last 5 years with the publication of new data in animal models and in other related autoimmune diseases. In contrast to other autoimmune diseases, we have less scientific evidences of direct relationship between presence of commensal microorganisms and SLE pathogenesis. One of the first evidences of this potential association was the isolation of *Helicobacter pylori* in the 67% of kidney biopsies from a cohort of the SLE-related disease LN (25). Apart from this isolated case, most of the microbial evidence in human SLE derives from animal models and *in vitro* experiments (26). These evidences of the involvement of gut microbiota on SLE and SLE-related autoimmune diseases are represented in Figure 1.

## Influence of Commensal Microbiota on Autoimmune Diseases I: Molecular Mimicry

The potential importance of gut bacteria in SLE may be deduced from the pristane-induced animal model (27). Pristane, or 2,6,10,14-tetramethylpentadecane, is a saturated alkane present in shark liver oil able to induce an autoimmune disease similar to SLE in mice (28). Pristane administration was associated with different degrees of hypergammaglobulinemia in conventional housed and microbiota-free mice; more concisely, presence of gut microbiota was associated with lower IgM but higher IgA and IgG titers. Interestingly, production of autoantibodies was markedly lower and delayed in microbiota-free mice with respect to control mice, suggesting the existence of a microbiota-triggered stimulus in this murine SLE model (27).

Some microbial structures have the ability to activate autoreactive T cells in certain host genetic backgrounds prone to autoimmunity. For instance, both bacterial-DNA and different cell-wall components, induced the production of anti-double-stranded DNA (dsDNA) autoantibodies in mice (29). Molecular mimicry has been hypothesized as the mechanism connecting the infection of *Burkholderia* sp. and the exacerbation of SLE symptoms (30). Molecular mimicry occurs when an antibody raised against a given antigen is able to recognize another antigen from a different molecule, for instance, two different amino acid epitopes coming from two proteins (31). In the framework of autoimmune diseases, antibodies developed against bacterial antigens during infection are supposed to recognize self-antigens, inducing formation of immune complexes that contribute, for instance, to renal damages. On the contrary, certain bacterial molecules may activate autoreactive T cells.

There are several examples suggesting an involvement of molecular mimicry in SLE-related diseases (Table 1). Antibodies developed against dsDNA were shown to bind to the sequence ARVLWRATH from cytochrome B 561 and to sequence RAGTDEGFG from one of the transcription regulators from *Burkholderia* sp. (30). Another example has been documented in an autoimmune disease-denominated glomerulonephritis. In this case, the amino acid residues 72–80 of the bacterial adhesin FimH and the human lysosomal-associated membrane protein (hLAMP-2) are recognized by autoantibodies (32). FimH is a fimbria subunit that is present in Gram-negative enteropathogens, among which species from the genus *Escherichia*, *Klebsiella*, and *Proteus*. Infections by Gram-negative bacteria can be positioned just before glomerulonephritis onset, as well as before detection of autoantibodies raised against hLAMP-2 (33), suggesting a potential role of a bacterial infection with the generation of autoantibodies and with the onset of the disease. Molecular mimicry may also be the mechanism underlying the activation of the T-cell population reactive against certain SLE antigens such as Ro60, a protein binding to small, non-coding RNAs termed “Y RNAs” (34). Although authors claimed that multiple commensal microbial peptides are able to activate T cells reactive against Ro60, no further information on the amino acid sequences is available.

**Table 1:** **Molecular mimicry between molecules of bacterial origin and host structures**.

Bacterial species	Bacterial molecule	Epitope	Host target
*Burkholderia* sp.	Cytochrome B 561	ARVLWRATH	dsDNA
*Burkholderia* sp.	Transcription regulator	RAGTDEGFG	dsDNA
*Escherichia* sp.	FimH	Residues 72–80	hLAMP-2
*Klebsiella* sp.	FimH	Residues 72–80	hLAMP-2
*Proteus* sp.	FimH	Residues 72–80	hLAMP-2
–	–	Microbial peptides	Ro60
*Campylobacter jejuni*	Lipoligosaccharides	–	Gangliosides
*Haemophilus influenzae*	Surface proteins	TLRVYK	β2-glycoprotein 1
*Neisseria gonorrhoeae*	Surface proteins	TLRVYK	β2-glycoprotein 1
Tetanus toxoid	Surface proteins	TLRVYK	β2-glycoprotein 1
*Streptococcus pneumoniae*	Exopolysaccharide	–	dsDNA, histones, renal proteins
*Escherichia coli*	Genomic DNA	CpG islands	dsDNA
*Vibrio cholerae*	Cholera toxin B	–	dsDNA

Molecular mimicry involving bacterial molecules is also involved in the generation of autoantibodies in other autoimmune diseases, such as antiphospholipid syndrome (APS) (16). In this disease, certain surface molecules such as lipoligosaccharides from *Campylobacter jejuni* are believed to mimic gangliosides from the human nerves, generating and maintaining the production of autoantibodies. Notably, certain bacterial sequences mimic the self-targets of aPLs autoantibodies, such as TLRVYK sequence from β2-glycoprotein 1, which shows homology to surface proteins from *Haemophilus influenzae*, *Neisseria gonorrhoeae*, or the tetanus toxoid (16). Several antibodies developed against pneumococcal antigens showed cross-reactivity against self-antigens, notably dsDNA (35). Eight out of those antibodies were also able to recognize bacterial exopolysaccharide, DNA, and histones, as well as glomerular structures such as some renal proteins (36). Additional evidence of molecular mimicry underlying bacterial infections are the antibodies raised against CpG DNA of *Escherichia coli* and the cholera toxin B of *Vibrio cholerae*, which promoted production of DNA autoantibodies in mice models (37).

## Influence of Commensal Microbiota on Autoimmune Diseases II: Superantigens

Superantigens are a class of toxins produced by many bacteria and viruses, which have the capacity to massively activate immune cells, by binding simultaneously to major histocompatibility complex class II proteins on antigen-presenting cells, and to the specific T-cell receptors on activated T cells (38). Superantigens are involved, among other pathological processes such as bacterial toxic shocks, in inducing autoimmunity by activating self-reactive T cells. Different bacterial molecules act as antigens and are involved in the trigger and progression of many autoimmune diseases. For instance, relapses of granulomatosis, an autoimmune disorder affecting kidneys, is directly correlated with a previous infection of superantigen producing *Staphylococcus aureus* strains in the upper respiratory tract (39). In this process, the adhesion properties of the staphylococcal acid phosphatase have a pivotal role through its binding to endothelial cells (40).

It should be mentioned that although molecular mimicry and superantigen involvement are well-explained in animal models, further research is needed in order to identify specific interactions between microbiota and immune system responsible for the generation and maintenance of autoreactive T cells in the human host, as well as for the production of autoantibodies.

## Metagenomic Studies Show an Intestinal Dysbiosis Associated with SLE

During the last years, high-throughput technologies have made it possible to deeply characterize the composition of the microbial populations inhabiting our gut, and to associate certain composition patterns, or the absence/presence of certain species with different disorders. One of the first *in vivo* studies, suggesting a relationship between alterations in the gut microbiota and a female-biased autoimmune disease, was published 4 years ago using a model of type 1 diabetes, the non-obese diabetic (NOD) mice (it should be noted that in humans the type 1 diabetes is not sex-biased) (18, 19). In this work, gut colonization by the Th17 inducing SFB was associated with the absence of development of type 1 diabetes. Moreover, knock out lines of the NOD mice lacking the myeloid differentiation primary response gene (MyD88), which is an adaptor molecule downstream the signaling pathway of certain toll-like receptors (TLRs), resulted in changes on the microbiota composition and conferred a protective effect on type 1 diabetes (41). These results strongly suggested a role for the microbiota composition in the context of female-biased autoimmune diseases, such as SLE or the APS.

In the moment where this manuscript was drafted, two metagenomic studies concerning SLE were available, one in the murine MRL/lpr model and the other in humans (1, 2, 42). From a microbial point of view, the situation is slightly different in humans when compared to mice. MRL/lpr mouse females showed higher numbers of the families *Lachnospiraceae* and *Bacteroidetes*, and lower numbers of *Bifidobacteriaceae* and *Erysipelotrichaceae* (42). Overall, the human situation was summarized by decreases on the Firmicutes/Bacteroidetes ratio in the samples corresponding to SLE patients (1, 2). Decreases in this ratio have also been observed in human type-2 diabetes and in Crohn’s disease when compared to control populations (43, 44).

Being more precise, pronounced increases in the Bacteroidetes/Prevotellaceae groups, and decreases of the Lachnospiraceae/Ruminococcaceae members were observed in the human SLE cohort (1, 2). Some of the species belonging to the Lachnospiraceae family comprise butyrate producers, such as *Roseburia* sp. or *Butyrivibrio* sp. Butyrate production in the human gut is relevant since it promotes differentiation of Tregs, a T-cell type able to suppress any kind of pro-inflammatory response (45).

Whereas the same observations were reported for Bacteroidetes populations in mice, Lachnospiraceae and Ruminococcaceae families showed opposite behaviors. This might be a direct consequence of the mutation carried by MRL/lpr mice (Fas^lpr^), which makes them deficient in Fas-mediated signaling. This genetic deficiency makes T cells refractory to butyrate-induced apoptosis, and this in turn may have a consequence in the composition of the intestinal microbiota (46). Decreases in members of the family Lachnospiraceae have been proposed as a parameter for monitoring disease activity in inflammatory bowel disease, and it remains to be validated whether this can also be applied to SLE (47). For this purpose, further metagenomic studies, involving larger cohorts, will be needed.

## Influence of Microbial DNA through Toll-Like Receptors 7 and 9

One of the factors that has delayed the study of the microbial influence on SLE pathogenesis was the absence of correlation between symptoms and breeding conditions in murine models, as this strongly affect the microbial load of the animals (4). Some scientific studies performed in the last years suggested an important role for two receptors of the innate immune system, TLR7 and TLR9, in SLE pathogenesis; the involvement of TLR4 being also under investigation (48). TLRs recognize specific microorganism ligands, and are one of the first lines of defense against external threats. Among the 10 TLRs identified so far in humans (12 in mice), TLR7 recognizes single-stranded viral RNA (ssRNA), whereas TLR9 recognizes both bacterial/viral dsDNA. The main TLR4 ligand is LPS, but it can also recognize different proteins such as viral glycoproteins, heat-shock proteins, and fibronectin, among others.

Expression of both TLR7/9 genes is increased in SLE patients when compared to healthy controls and this expression level correlates positively with levels of typical serological pro-inflammatory markers, such as IL-6, IFN-gamma, and TNF-alpha (49). Self-RNA and self-DNA are main targets for autoantibody generation in autoimmune diseases, and the complex nucleic acid – antibody seems to contribute, through an altered recognition by TLR7 and TLR9, to the aberrant immune responses observed in SLE (50). This includes activation and expansion of autoreactive B and T cells (51); in fact, triggering of autoreactive memory B cells by bacterial/viral DNA through the action of TLR9 has been proposed to be implicated in SLE relapses (52).

Results from animal models have shown that alterations of the innate immune system through the action of specific receptors, such as TLRs, correlate with SLE symptoms. Duplication of TLR7 gene in a SLE mouse model (Yaa mice), has been shown to be directly related to the disease by an excessive auto-RNA signaling through this receptor (53). In fact, the number of TLR7 molecules is directly related to the risk of the development of SLE in mice, supporting the role of the innate immune system in the onset of this disease. Duplication or overexpression of the *tlr*7 gene resulted in increased titters of RNA-specific autoantibodies, whereas deficiencies in the TLR9 signalization pathway involved increased production of anti-DNA/anti-chromatin antibodies (54).

## A Role for the Virome in SLE?

An important part of the microbial populations inhabiting our body are virus and bacteriophages, which are referred collectively as the human virome. The first evidence suggesting that viruses were involved in the triggering and development of SLE was the higher IFN-α levels found in the blood of a SLE cohort (55). Infection by Epstein–Barr virus (EBV) has been correlated in LN through a higher production of anti-Sm antibodies, which are autoantibodies directed against seven proteins of different ribonucleoproteins (56). Certain experimental studies indicated a connection between an abnormal immune response to EBV and SLE; more concisely, it has been observed that SLE patients developed specific anti-EBV IgG antibodies that were absent in healthy controls, and vice versa (57). From an epidemic point of view, EBV infection is associated with a higher risk of developing SLE and other autoimmune diseases, such as multiple sclerosis (58). As EBV infects B cells (and other immune cells) inducing the production of pro-inflammatory cytokines, this fact and an aberrant immune response to this virus could be linked to SLE. Further research will elucidate the precise role of viruses in SLE.

## Diet, Microbiota, and SLE

From a nutrition point of view, most of the work in SLE has been traditionally focused on the immunomodulatory effect of single dietary components with a demonstrated role on the immune system. Although very little is known about the impact of diet on microbiota in SLE subjects, there is some scientific evidence supporting the hypothesis that some of the dietary impact on SLE pathology could be achieved through modulation of the gut microbiota (4). Currently, there is not enough information to estimate if a dietary intervention could work on preventing SLE relapses. However, evidence from other diseases with an associated dysbiosis showed the impact of several dietary components in balancing certain microorganism populations, which may be applied to SLE.

It has been reported that a diet restricted in carbohydrates or fat, balanced the gut microbiota increasing the proportion of *Bacteroidetes* in obese subjects with high *Firmicutes/Bacteroidetes* ratios (compared to lean controls) (59). In this sense, Western diets, characterized by their richness in animal proteins and saturated fats, have also been correlated with a greater abundance of *Bacteroidetes* (60). On the contrary, diets supplemented with whole grains produced an increase in *Firmicutes/Bacteroidetes* ratio (61), and the administration of a low-fat/high-fiber diet has been correlated positively with *Firmicutes* (62). In consonance with these findings, lower levels of *Bacteroidetes* were observed in African children with a long-term consumption of diets rich in whole grains, dietary fibers, and vegetables supporting the health benefits of whole grains by means of microbiota modulation (63).

To the best of our knowledge, there is no intervention study in the literature to test the effects of the amount and type of fats included in diet on SLE microbiota. A descriptive work from our research group did not support the association between fats or saturate fatty acids at moderate amounts in a well-balanced diet and fecal microbiota in a SLE cohort (64). It is noteworthy that some of the effects ascribed to fat intake are dose- and food-dependent, i.e., they show different effects depending on the food where they are found. For instance, presence of orange juice in a high-fat meal decreased the impact of fats on oxidative stress (65). For this reason, some authors have suggested studying the interactions between the different components of a meal, rather to focus on the effects of a single ingredient (66).

Recently, we have reported on the association between the intake of fruits, such as oranges and apples, and decreases in specific microorganism populations in SLE patients (64). It is important to consider that these fruits are natural dietary sources of polyphenols and fibers with a high potential to modulate microbiota. Specific phenolic compounds like dihydrochalcones from apples, along with dietary fiber, are degraded by bifidobacteria promoting its growth (64, 67, 68). These kinds of changes could be of special interest for SLE patients given the immunomodulatory effect attributed to some strains of the genus *Bifidobacterium* (69). For instance *Bifidobacterium bifidum* LMG13195 strain promoted the expansion of T_reg_ cells, a T-cell population favoring mucosal homeostasis (70, 71). Other works showed positive associations between dietary flavones and the levels of *Blautia* sp., a member of the *Clostridium* cluster XIVa. As this microorganism is also involved in the expansion of T_reg_ cells, promoting its growth through the diet may be interesting in order to maintain/restore immune homeostasis (72).

Finally, it is very interesting to mention the effect of vitamin A in the diet. A dietary intervention with vitamin A in animal models has returned improvements of the SLE pathogenesis, notably in the reduction of both proteinuria and glomerulonephritis, and has been shown to globally reduce the LN symptoms (73, 74). Another dietary intervention study showed that the intake of retinoic acid, which is the active form of vitamin A (retinol), restored the lactobacilli levels that were decreased during SLE development in mice (42). This is not the only known effect of vitamin A on our gut commensals, as the growth of SFB is influenced by the levels of this vitamin, and as stated before this bacterial group appears to play crucial roles in the development of several SLE-like diseases through the expansion of the Th17 cell subset (4).

## Perspectives

**Figure 2:**
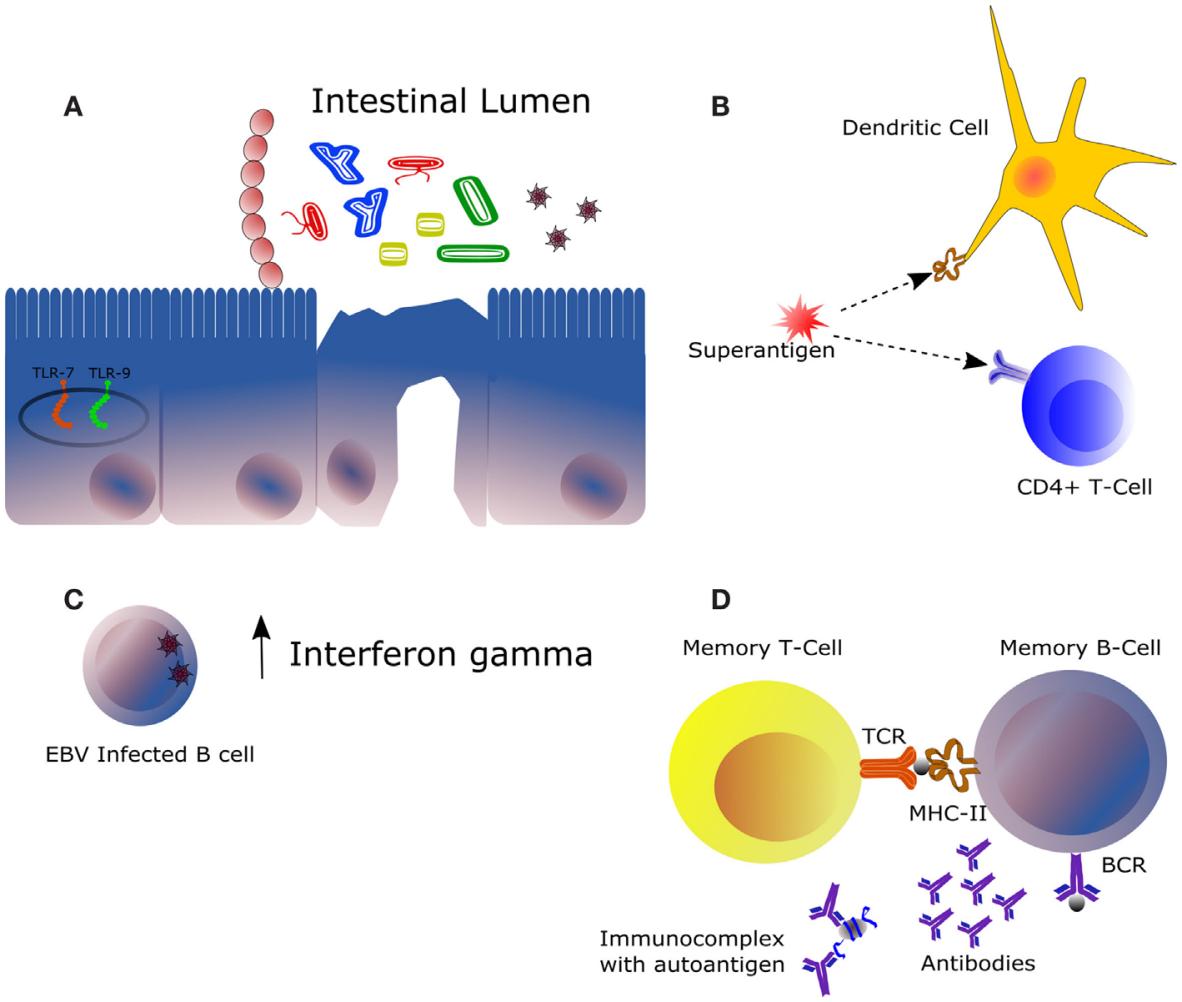
**Main mechanisms of potential influence of the intestinal microbiota in the framework of autoimmune diseases**. **(A)** The immunomodulatory effect of the intestinal microbiota is highly dependent on its composition and on the immunological status of the host. Some bacteria trigger downstream effector responses, such as Th1, Th17, or Th2, whereas other acts by expanding the subset of regulatory T cells. Expression of certain receptors recognizing bacterial and viral DNA, mainly TLR7 and TLR9, is increased in the context of SLE. **(B)** Superantigens are molecules activating both innate (represented by a dendritic cell) and adaptative immunity (represented by a T-cell clone), being this the mechanism of action of some pathogens notably at the level of the upper respiratory tract. **(C)** Infection of the Epstein–Barr virus in B cells could be responsible for the increased levels of interferon gamma observed in the serum of certain SLE patients. IFNγ is produced mainly by natural killer and natural killer T cells during innate immune response and by CD4^+^ Th1 and CD8^+^ cytotoxic T lymphocyte cells during adaptative immune response. **(D)** Autoreactive B and T cells may be activated by antigens from bacterial origin leading to production of auto antibodies. This similarity between certain bacteria antigens, such as peptides (represented by the gray sphere) and autoantigens at the molecular level, is denominated molecular mimicry. Clearance of immune complexes is directly related to damages at the kidney level. In a context of autoimmunity, increased production of autoantibodies involves higher formation of such complexes.

In this review, we have shown that gut bacteria composition may have a role in SLE as deduced from SLE-related autoimmune diseases, female-biased or not. Potential mechanisms involved are summarized in Figure 2. With the incoming of novel metagenomic and metatranscriptomic studies focused on SLE microbiota, we will undoubtedly describe the precise role of the different microbial population on SLE and other autoimmune diseases. Altered microbial colonization in the gut (such as abnormal Firmicutes:Bacteroidetes ratio) might contribute to a misbalanced immune response responsible for the triggering or the relapses of the disease. In this sense, it might be hypothesized that excessive Bacteroides signaling through our innate immune system might have a role in SLE pathogenesis, although this is a speculative statement that deserves further investigation.

Scientific results evidenced how some surface-associate proteins and DNA/RNA from both bacteria and viruses are wrongly recognized by the immune system. This molecular mimicry lead autoreactive immune cells to attack self-structures in response to bacterial exposure. Therefore, future research will elucidate whether SLE patients also develop antibodies against the commensal microbiota, and if molecules derived from are able to activate autoreactive T cells through molecular mimicry (1, 2).

Dietary interventions will undoubtedly be of great importance to generate new designs of functional foods focused to correct dysbiosis observed in SLE and other autoimmune diseases. For instance, administration of specific commensals, used as probiotics, might be designed to specifically treat SLE. Use of microorganisms can be dual, as they are able to both displace undesired populations and modulate host immune responses. In this sense, the usefulness of experimental treatments such as fecal microbiota transplantation separated and normalized from the rest of feces components through density gradients, or application of immunomodulatory peptides obtained from our commensal microbiota might be important in the future.

## Author Contributions

BS and AM conceived the manuscript. BS, AH, and SG drafted the manuscript and all the authors revised the final version prior submission.

## Conflict of Interest Statement

The authors declare that the research was conducted in the absence of any commercial or financial relationships that could be construed as a potential conflict of interest.

## References

[1] Hevia A Lopez P Suarez A Jacquot C Urdaci MC Margolles A Association of levels of antibodies from patients with inflammatory bowel disease with extracellular proteins of food and probiotic bacteria. Biomed Res Int (2014) 2014:1–8. 10.1155/2014/351204 24991549 PMC4065772

[2] Hevia A Milani C López P Cuervo A Arboleya S Duranti S Intestinal dysbiosis associated with systemic lupus erythematosus. MBio (2014) 5:e1548–1514. 10.1128/mBio.01548-14 25271284 PMC4196225

[3] Hill C Guarner F Reid G Gibson GR Merenstein DJ Pot B The International Scientific Association for Probiotics and Prebiotics consensus statement on the scope and appropriate use of the term probiotic. Nat Rev Gastroenterol Hepatol (2014) 11:506–14. 10.1038/nrgastro.2014.66 24912386

[4] Vieira SM Pagovich OE Kriegel MA Diet, microbiota and autoimmune diseases. Lupus (2014) 23:518–26. 10.1177/0961203313501401 24763536 PMC4009622

[5] Ruiz L Hevia A Bernardo D Margolles A Sanchez B. Extracellular molecular effectors mediating probiotic attributes. FEMS Microbiol Lett (2014) 359:1–11. 10.1111/1574-6968.12576 25115731

[6] Mok CC Lau CS. Pathogenesis of systemic lupus erythematosus. J Clin Pathol (2003) 56:481–90. 10.1136/jcp.56.7.481 12835292 PMC1769989

[7] Sherer Y Gorstein A Fritzler MJ Shoenfeld Y. Autoantibody explosion in systemic lupus erythematosus: more than 100 different antibodies found in SLE patients. Semin Arthritis Rheum (2004) 34:501–37. 10.1016/j.semarthrit.2004.07.002 15505768

[8] Santiago-Raber ML Borel P Uematsu S Akira S Izuil S. Role of TLR7 and TLR9 in a murine model of SLE. Swiss Med Wkly (2008) 138:15s–15s. 10.1186/ar2771 19624844 PMC2745794

[9] Hochberg MC Updating the American college of rheumatology revised criteria for the classification of systemic lupus erythematosus. Arthritis Rheum (1997) 40:1725–1725. 10.1002/art.1780400928 9324032

[10] Cambridge G Leandro MJ Isenberg DA Teodorescu M Ehrenstein MR Edwards JCW. B cell depletion therapy in systemic lupus erythematosus: effect on autoantibody and anti-microbial antibody profiles. Arthritis Rheum (2004) 50:S227–8. 10.1002/art.22211 17075806

[11] Cambier JC. Autoimmunity risk alleles: hotspots in B cell regulatory signaling pathways. J Clin Investig (2013) 123:1928–31. 10.1172/JCI69289 23619359 PMC3635745

[12] Apperloorenkema HZ Bootsma H Mulder BI Kallenberg CGB Vanderwaaij D. Host-microflora interaction in systemic lupus-erythematosus (Sle) – circulating antibodies to the indigenous bacteria of the intestinal-tract. Epidemiol Infect (1995) 114:133–41. 10.1017/S0950268800051980 7867731 PMC2271335

[13] Edwards CJ Costenbader KH Epigenetics and the microbiome: developing areas in the understanding of the aetiology of lupus. Lupus (2014) 23:505–6. 10.1177/0961203314531636 24763534

[14] Markle JG Fish EN. SeXX matters in immunity. Trends Immunol (2014) 35:97–104. 10.1016/j.it.2013.10.006 24239225

[15] Zhang HS Sparks JB Karyala SV Settlage R Luo XM. Host adaptive immunity alters gut microbiota. ISME J (2015) 9:770–81. 10.1038/ismej.2014.165 25216087 PMC4331585

[16] Ruff WE Vieira SM Kriegel MA. The role of the gut microbiota in the pathogenesis of antiphospholipid syndrome. Curr Rheumatol Rep (2015) 17:472. 10.1007/s11926-014-0472-1 25475595 PMC4394866

[17] Wu HJ Ivanov II Darce J Hattori K Shima T Umesaki Y Gut-residing segmented filamentous bacteria drive autoimmune arthritis via T helper 17 cells. Immunity (2010) 32:815–27. 10.1016/j.immuni.2010.06.001 20620945 PMC2904693

[18] Kriegel MA Sefik E Hill JA Wu HJ Benoist C Mathis D Sex-specific effects of segmented filamentous bacteria in the autoimmune-prone NOD mouse strain-segregation with diabetes protection in females but not males. Arthritis Rheum (2011) 63:S918–918. 10.1002/art.33310

[19] Kriegel MA Sefik E Hill JA Wu HJ Benoist C Mathis D. Naturally transmitted segmented filamentous bacteria segregate with diabetes protection in nonobese diabetic mice. Proc Natl Acad Sci U S A (2011) 108:11548–53. 10.1073/pnas.1108924108 21709219 PMC3136249

[20] Cantwell AR Kelso DW Jones JE. Histologic observations of coccoid forms suggestive of cell-wall deficient bacteria in cutaneous and systemic lupus-erythematosus. Int J Dermatol (1982) 21:526–37. 10.1111/j.1365-4362.1982.tb01198.x 6759425

[21] Gilkeson GS Grudier JP Karounos DG Pisetsky DS. Induction of anti-double stranded DNA antibodies in normal mice by immunization with bacterial-DNA. J Immunol (1989) 142:1482–6. 2645362

[22] Granholm NA Cavallo T. Long-lasting effects of bacterial lipopolysaccharide promote progression of lupus nephritis in Nzb/W mice. Lupus (1994) 3:507–14. 10.1177/096120339400300614 7704009

[23] Fournie GJ Lambert PH Miescher PA. Release of DNA in circulating blood and induction of anti-DNA antibodies after injection of bacterial lipopolysaccharides. J Exp Med (1974) 140:1189–206. 10.1084/jem.140.5.1189 4607609 PMC2139721

[24] Izui S Lambert PH Fournie GJ Turler H Miescher PA. Features of systemic lupus-erythematosus in mice injected with bacterial lipopolysaccharides – identification of circulating DNA and renal localization of DNA-anti-DNA complexes. J Exp Med (1977) 145:1115–30. 10.1084/jem.145.5.1115 323400 PMC2180666

[25] Li Q Lin X Wu Z He L Wang W Cao Q Immuno-histochemistry analysis of *Helicobacter pylori* antigen in renal biopsy specimens from patients with glomerulonephritis. Saudi J Kidney Dis Transpl (2013) 24:751–8. 10.4103/1319-2442.113871 23816725

[26] Kronbichler A Kerschbaum J Mayer G. The influence and role of microbial factors in autoimmune kidney diseases: a systematic review. J Immunol Res (2015) 2015:858027. 10.1155/2015/858027 26078982 PMC4452370

[27] Hamilton KJ Satoh M Swartz J Richards HB Reeves WH. Influence of microbial stimulation on hypergammaglobulinemia and autoantibody production in pristane-induced lupus. Clin Immunol Immunopathol (1998) 86:271–9. 10.1006/clin.1997.4481 9557160

[28] Perry D Sang A Yin YM Zheng YY Morel L. Murine models of systemic lupus erythematosus. J Biomed Biotechnol (2011) 2011:1–19. 10.1155/2011/271694 21403825 PMC3042628

[29] Hahn BH Antibodies to DNA. N Engl J Med (1998) 338:1359–68. 10.1056/NEJM199805073381906 9571257

[30] Zhang W Reichlin M. A possible link between infection with burkholderia bacteria and systemic lupus erythematosus based on epitope mimicry. Clin Dev Immunol (2008) 2008:1–7. 10.1155/2008/683489 18682819 PMC2494591

[31] Oldstone MBA. Molecular mimicry and immune-mediated diseases. FASEB J (1998) 12:1255–65. 9761770 10.1096/fasebj.12.13.1255 PMC7164021

[32] Kain R Exner M Brandes R Ziebermayr R Cunningham D Alderson CA Molecular mimicry in pauci-immune focal necrotizing glomerulonephritis. Nat Med (2008) 14:1088–96. 10.1038/nm.1874 18836458 PMC2751601

[33] Roth AJ Brown MC Smith RN Badhwar AK Parente O Chung Hc Anti-LAMP-2 antibodies are not prevalent in patients with antineutrophil cytoplasmic autoantibody glomerulonephritis. J Am Soc Nephrol (2012) 23:545–55. 10.1681/ASN.2011030273 22021709 PMC3294309

[34] Szymula A Szczerba B Bagavant H Fu SM Deshmukh U A case for microbial involvement in the activation of lupus-antigen reactive T cells. J Immunol (2012) 188:171–39.

[35] Kowal C Weinstein A Diamond B. Molecular mimicry between bacterial and self antigen in a patient with systemic lupus erythematosus. Eur J Immunol (1999) 29:1901–11. 10.1002/(SICI)1521-4141(199906)29:06<1901::AID-IMMU1901>3.0.CO;2-L 10382752

[36] Chowdhry IA Kowal C Hardin J Zhou Z Diamond B. Autoantibodies that bind glomeruli: cross-reactivity with bacterial antigen. Arthritis Rheum (2005) 52:2403–10. 10.1002/art.21143 16052539

[37] Deng GM Tsokos GC. Cholera toxin B accelerates disease progression in lupus-prone mice by promoting lipid raft aggregation. J Immunol (2008) 181:4019–26. 10.4049/jimmunol.181.6.4019 18768857 PMC2556981

[38] Kearney DE Wang W Redmond HP Wang JH. Bacterial superantigens enhance the in vitro proinflammatory response and in vivo lethality of the TLR2 agonist bacterial lipoprotein. J Immunol (2011) 187:5363–9. 10.4049/jimmunol.1003747 22003201

[39] Popa ER Stegeman CA Abdulahad WH van der Meer B Arends J Manson WM Staphylococcal toxic-shock-syndrome-toxin-1 as a risk factor for disease relapse in Wegener’s granulomatosis. Rheumatology (2007) 46:1029–33. 10.1093/rheumatology/kem022 17409134

[40] Brons RH Bakker HI Van Wijk RT Van Dijk NW Kobold ACM Limburg PC Staphylococcal acid phosphatase binds to endothelial cells via charge interaction; a pathogenic role in Wegener’s granulomatosis? Clin Exp Immunol (2000) 119:566–73. 10.1046/j.1365-2249.2000.01172.x 10691932 PMC1905582

[41] Wen L Ley RE Volchkov PY Stranges PB Avanesyan L Stonebraker AC Innate immunity and intestinal microbiota in the development of type 1 diabetes. Nature (2008) 455:U1109–1110. 10.1038/nature07336 18806780 PMC2574766

[42] Zhang HS Liao XF Sparks JB Luo XM. Dynamics of gut microbiota in autoimmune lupus. Appl Environ Microbiol (2014) 80:7551–60. 10.1128/AEM.02676-14 25261516 PMC4249226

[43] Larsen N Vogensen FK van den Berg FW Nielsen DS Andreasen AS Pedersen BK Gut microbiota in human adults with type 2 diabetes differs from non-diabetic adults. PLoS One (2010) 5:e9085. 10.1371/journal.pone.0009085 20140211 PMC2816710

[44] Man SM Kaakoush NO Mitchell HM. The role of bacteria and pattern-recognition receptors in Crohn’s disease. Nat Rev Gastroenterol Hepatol (2011) 8:152–68. 10.1038/nrgastro.2011.3 21304476

[45] Singh N Gurav A Sivaprakasam S Brady E Padia R Shi H Activation of Gpr109a, receptor for niacin and the commensal metabolite butyrate, suppresses colonic inflammation and carcinogenesis. Immunity (2014) 40:128–39. 10.1016/j.immuni.2013.12.007 24412617 PMC4305274

[46] Zimmerman MA Singh N Martin PM Thangaraju M Ganapathy V Waller JL Butyrate suppresses colonic inflammation through HDAC1-dependent Fas upregulation and Fas-mediated apoptosis of T cells. Am J Physiol Gastrointest Liver Physiol (2012) 302:G1405–15. 10.1152/ajpgi.00543.2011 22517765 PMC3378095

[47] Berry D Reinisch W. Intestinal microbiota: a source of novel biomarkers in inflammatory bowel diseases? Best Pract Res Clin Gastroenterol (2013) 27:47–58. 10.1016/j.bpg.2013.03.005 23768552

[48] Richez C Blanco P Rifkin I Moreau JF Schaeverbeke T. Role for toll-like receptors in autoimmune disease: the example of systemic lupus erythematosus. Joint Bone Spine (2011) 78:124–30. 10.1016/j.jbspin.2010.09.005 20961794

[49] Lyn-Cook BD Xie C Oates J Treadwell E Word B Hammons G Increased expression of toll-like receptors (TLRs) 7 and 9 and other cytokines in systemic lupus erythematosus (SLE) patients: ethnic differences and potential new targets for therapeutic drugs. Mol Immunol (2014) 61:38–43. 10.1016/j.molimm.2014.05.001 24865418

[50] Celhar T Magalhes R Fairhurst AM. TLR7 and TLR9 in SLE: when sensing self goes wrong. Immunol Res (2012) 53:58–77. 10.1007/s12026-012-8270-1 22434514

[51] Kessel A Peri R Haj T Snir A Slobodin G Sabo E IVIg attenuates TLR-9 activation in B cells from SLE patients. J Clin Immunol (2011) 31:30–8. 10.1007/s10875-010-9469-3 20922561

[52] Capolunghi F Rosado MM Cascioli S Girolami E Bordasco S Vivarelli M Pharmacological inhibition of TLR9 activation blocks autoantibody production in human B cells from SLE patients. Rheumatology (2010) 49:2281–9. 10.1093/rheumatology/keq226 20739362

[53] Pisitkun P Deane JA Difilippantonio MJ Tarasenko T Satterthwaite AB Bolland S Genetic modifiers of SLE and Btk-dependent Anti-RNA B cells in Yaa mice due to TLR7 gene duplication. Arthritis Rheum (2006) 54:S776–776.

[54] Santiago-Raber ML Baudino L Izui S. Emerging roles of TLR7 and TLR9 in murine SLE. J Autoimmun (2009) 33:231–8. 10.1016/j.jaut.2009.10.001 19846276

[55] Lichtman EI Helfgott SM Kriegel MA. Emerging therapies for systemic lupus erythematosus – focus on targeting interferon-alpha. Clin Immunol (2012) 143:210–21. 10.1016/j.clim.2012.03.005 22525889 PMC3358492

[56] Yu XX Yao CW Tao JL Yang C Luo MN Li SM The expression of renal Epstein-Barr virus markers in patients with lupus nephritis. Exp Ther Med (2014) 7:1135–40. 10.3892/etm.2014.1578 24940399 PMC3991543

[57] Fattal I Shental N Molad Y Gabrielli A Pokroy-Shapira E Oren S Epstein-Barr virus antibodies mark systemic lupus erythematosus and scleroderma patients negative for anti-DNA. Immunology (2014) 141:276–85. 10.1111/imm.12200 24164500 PMC3904248

[58] Casiraghi C Horwitz MS Epstein-Barr virus and autoimmunity: the role of a latent viral infection in multiple sclerosis and systemic lupus erythematosus pathogenesis. Future Virol (2013) 8:173–82. 10.2217/fvl.12.136

[59] Ley RE Turnbaugh PJ Klein S Gordon JI. Microbial ecology – human gut microbes associated with obesity. Nature (2006) 444:1022–3. 10.1038/4441022a 17183309

[60] Yatsunenko T Rey FE Manary MJ Trehan I Dominguez-Bello MG Contreras M Human gut microbiome viewed across age and geography. Nature (2012) 486:222–7. 10.1038/nature11053 22699611 PMC3376388

[61] Martínez I Lattimer JM Hubach KL Case JA Yang J Weber CG Gut microbiome composition is linked to whole grain-induced immunological improvements. ISME J (2013) 7:269–80. 10.1038/ismej.2012.104 23038174 PMC3554403

[62] Wu GD Chen J Hoffmann C Bittinger K Chen YY Keilbaugh SA Linking long-term dietary patterns with gut microbial enterotypes. Science (2011) 334:105–8. 10.1126/science.1208344 21885731 PMC3368382

[63] De Filippo C Cavalieri D Di Paola M Ramazzotti M Poullet JB Massart S Impact of diet in shaping gut microbiota revealed by a comparative study in children from Europe and rural Africa. Proc Natl Acad Sci U S A (2010) 107:14691–6. 10.1073/pnas.1005963107 20679230 PMC2930426

[64] Cuervo A Hevia A Lopez P Suarez A Sanchez B Margolles A Association of polyphenols from oranges and apples with specific intestinal microorganisms in systemic lupus erythematosus patients. Nutrients (2015) 7:1301–17. 10.3390/nu7021301 25690419 PMC4344589

[65] Ghanim H Sia CL Upadhyay M Korzeniewski K Viswanathan P Abuaysheh S Orange juice neutralizes the proinflammatory effect of a high-fat, high-carbohydrate meal and prevents endotoxin increase and toll-like receptor expression. Am J Clin Nutr (2010) 91:940–9. 10.3945/ajcn.2009.28584 20200256 PMC2844681

[66] Moreira APB Texeira TFS Ferreira AB Peluzio MDG Alfenas RDG. Influence of a high-fat diet on gut microbiota, intestinal permeability and metabolic endotoxaemia. Br J Nutr (2012) 108:801–9. 10.1017/S0007114512001213 22717075

[67] Sembries S Dongowski G Jacobasch G Mehrlander K Will F Dietrich H. Effects of dietary fibre-rich juice colloids from apple pomace extraction juices on intestinal fermentation products and microbiota in rats. Br J Nutr (2003) 90:607–15. 10.1079/BJN2003925 13129467

[68] Sembries S Dongowski G Mehrlander K Will F Dietrich H. Physiological effects of extraction juices from apple, grape, and red beet pomaces in rats. J Agric Food Chem (2006) 54:10269–80. 10.1021/jf0618168 17177570

[69] Konieczna P Akdis CA Quigley EM Shanahan F O’Mahony L. Portrait of an immunoregulatory *Bifidobacterium*. Gut Microbes (2012) 3:261–6. 10.4161/gmic.20358 22572827 PMC3427218

[70] Lopez P Gonzalez-Rodriguez I Gueimonde M Margolles A Suarez A. Immune response to *Bifidobacterium bifidum* strains support Treg/Th17 plasticity. PLoS One (2011) 6:e24776. 10.1371/journal.pone.0024776 21966367 PMC3178565

[71] Lopez P Gonzalez-Rodriguez I Sanchez B Ruas-Madiedo P Suarez A Margolles A Interaction of *Bifidobacterium bifidum* LMG13195 with HT29 cells influences regulatory-T-cell-associated chemokine receptor expression. Appl Environ Microbiol (2012) 78:2850–7. 10.1128/AEM.07581-11 22344636 PMC3318848

[72] Atarashi K Tanoue T Shima T Imaoka A Kuwahara T Momose Y Induction of colonic regulatory T cells by indigenous *Clostridium* species. Science (2011) 331:337–41. 10.1126/science.1198469 21205640 PMC3969237

[73] de Lema GP Lucio-Cazana FJ Molina A Luckow B Schmid H de Wit C Retinoic acid treatment protects MRL/lpr lupus mice from the development of glomerular disease. Kidney Int (2004) 66:1018–28. 10.1111/j.1523-1755.2004.00850.x 15327395

[74] Hsieh CC Lin BF. Dietary factors regulate cytokines in murine models of systemic lupus erythematosus. Autoimmun Rev (2011) 11:22–7. 10.1016/j.autrev.2011.06.009 21763466

